# Supplementary material for: Towards a comprehensive understanding of the structural dynamics of a bacterial diterpene synthase during catalysis
Source: Nat Commun. 2018 Sep 28;9:3971. doi: 10.1038/s41467-018-06325-8 (PMC6162201; doi:10.1038/s41467-018-06325-8)
Supplement: Supplementary file 1 — Supplementary Information [file 41467_2018_6325_MOESM1_ESM.pdf]

Supplementary Information

Towards a comprehensive understanding of the structural dynamics of a bacterial diterpene synthase during catalysis

Driller *et al.*

Supplementary Discussion

FGGDP co-crystallized with other TPSs

FGGDP has previously been co-crystallised with taxadiene synthase (TXS; PDB-ID 3P5R ref[1]), where FGGDP is bound in the active site with its diphosphate group still attached to the aliphatic tail. The reported TXS construct however was N-terminally truncated, stalling the enzyme in the catalytically inactive open conformation. Therefore, the mere binding of the substrate to the open TXS structure does not promote the complete closure of the active site. Consequently, FGGPP is not correctly positioned and cannot undergo cyclisation.

The non-canonical DDXD motif in comparison to other TPSs with the canonical DDXXD motif

The amino acid sequence of CotB2 shows an unconventional aspartate-rich ^{110}DDMD^{113} motif residing on helix D (Supplementary Fig. 11a). In the structure of CotB2^{wt}•$Mg^{2+}{}_3$•F-Dola D110 is directly involved in the coordination of $Mg^{2+}{}_A$ and $Mg^{2+}{}_C$, whereas D111 forms a salt-bridge with R294 of the RY-pair. D113 is surface exposed and points away from the active site (Supplementary Fig 11a and c). The difference in the protein sequence of the aspartate-rich motif has structural consequences (Supplementary Figure 11c). In the structure of epi-isozizaene synthase in complex with diphosphate, 3 Mg^{2+} ions and N-benzyl-N,N-diethylethanaminium,[2] with a canonical aspartate-rich motif ^{99}DDRHD^{103}, the α-helix carrying the motif is extended compared to CotB2 (Supplementary Fig. 11b). The first aspartate of the aspartate–rich motif resides at an identical position to coordinate $Mg^{2+}{}_A$ and $Mg^{2+}{}_C$. The second aspartate establishes as well a salt-bridge with the RY-pair. In contrast the third aspartate points towards the active site approaching water molecules in the water network around the Mg^{2+} ions.

Comparison of CotB2^{wt}•$Mg^{2+}{}_3$•F-Dola and CotB2^{wt}•$Mg^{2+}{}_B$•GGSDP

Tomita and colleagues stated they crystallised the fully closed structure of CotB2, the C-terminus ending with K296, hypothesising that the salt-bridge is the driving force of active site closure.[3] However, the remaining C-terminal residues have not been modelled due to their flexibility. In contrast, in

CotB2^{wt}•Mg^{2+}_3•F-Dola we observe the complete C-terminus, ending with the C-terminal Q307. This structural feature results in a higher rmsd between the open and closed structure (Supplementary Table 4), manifesting the importance of the C-terminus for proper closure of the enzyme. Since in CotB2^{wt}•Mg^{2+}_B•GGSDP two Mg^{2+}-ions are missing,[3] the diphosphate moiety is not properly coordinated (Fig. 2b), resulting in significant substrate flexibility within the catalytically active site. Moreover, in the reported structure the missing Mg^{2+} ions prevent helix D movement towards the active site thereby prohibiting folding over of the C-terminus. Interestingly, the hydrophobic tail of GGSDP adopts a position similar to the partly cyclised intermediate in our CotB2^{wt}•Mg^{2+}_3•F-Dola structure (Fig. 2b), as determined by the internal shape of the active site. In summary, our observations indicate that the crucial step of the initiation of the cyclisation reaction is the precise orientation and binding of the diphosphate moiety. The structure of CotB2^{wt}•Mg^{2+}_B•GGSDP on the other hand represents a pre-catalytic state of the enzyme reflecting a snapshot from the open to the fully closed conformation.

Supplementary Methods

Cloning. DNA manipulations and cloning procedures were performed according to standard protocols. The C-terminal deletion variant of CotB2 (CotB2$^{\Delta Cter}$) was designed by adding restriction sites *Nco*I at the 5'-end and *Hind*III at the 3'-end to the sequence (Supplementary Table 5; primer 1 and 2). The digested *Nco*I/*Hind*III fragment was ligated into the target-vector pETM-11 with a TEV-cleavable N-terminal hexa-histidine-tag. Single point mutations V80L, M189C, V150A, L281V and W288F of CotB2 were introduced by quick change site-directed mutagenesis (Supplementary Table 4; primer 3 to 12). Correctness of the amplified gene sequences was assessed by DNA sequencing.

Synthesis of (*2Z,6E,10E*)-2-Fluor-3,7,11,15-tetramethylhexadeca-2,6,10,14-tetraen-1-yl diphosphate (2-fluoro-geranylgeranyl diphosphate) tetrabutylammonium salt.

Materials were purchased from commercial suppliers and used without further purification. Synthesis was performed as described by Roe *et al.* [4] and some reactions were optimised.[5,6] The products of intermediate reaction steps were analysed by NMR and HRMS. The NMR spectra (Supplementary Figs. 8 and 9) and HRMS data (Supplementary Fig. 10) of the final product, 2-fluoro-geranylgeranyl diphosphate tetrabutylammonium salt, are presented below:

R$_f$ = 0.73 (*n*-Pentan)

^{1}H NMR (700 MHz, CD_3OD): δ = 5.15–5.03 (m, 3H, H-6, H-10, H-14), 4.65 (dd, *J* = 23.5, 5.3 Hz, 2H, H-1), 3.25–3.19 (m, 24H, 12xCH_2, NBu_4), 2.10–2.00 (m, 8H, H-4, H-5, H-9, H-13), 1.99–1.90 (m, 4H, H-8, H-12), 1.72 (d, *J* = 2.9 Hz, 3H, H-16), 1.69–1.60 (m, 24 H, 12xCH_2, NBu_4), 1.60–1.55 (m, 12H, H-17, H-18, H-19, H-20), 1.40 (tq, *J* = 14.8, 7.4 Hz, 24H, 12xCH_2, NBu_4), 1.00 (t, *J* = 7.4 Hz, 36H, 12xCH_3, NBu_4) ppm.

^{13}C NMR (175 MHz, CD_3OD): δ = 151.3 (d, *J* = 233.0 Hz, C-2), 135.1 (C-7 or C-11), 134.5 (C-7 or C-11), 130.7 (C-15), 124.1 (C-6 or C-10), 123.7 (C-6 or C-10), 117.2 (d, *J* = 15.8 Hz, C-3), 110.0 (C-14), 58.1 (d, *J* = 6.8 Hz, C-1), 39.5 (C-8 or C-12), 39.4 (C-8 or C-12), 29.7 (d, *J* = 7.1 Hz, C-4), 26.4 (C-5), 26.3 (C-9 orr C-13), 24.5 (C-9 or C-13), 23.4 (C-16), 19.3 (d, *J* = 3.5 Hz, C-17), 14.7 (C-17 or C-18 or C-19), 14.6 (C-17 or C-18 or C-19), 12.6 (C-17 or C-18 or C-19) ppm.

^{19}F NMR (376 MHz, CD_3OD): δ = –118.7 (t, J = 23.9 Hz) ppm.

^{31}P NMR (162 MHz, CD_3OD): δ = –6.8 (s, organic monophosphate), –8.9 (d, J = 19.6 Hz), –9.4 (d, J = 19.4 Hz) ppm.

HRMS (ESI-TOF, *m/z*): ($C_{20}H_{34}FO_7P_2^-$) $[M\text{-}H]^-$ = 467.1728 (calculated: 467.1768).

Bacterial strains, genes and vectors for diterpene production. Cloning, protein expression and production of cyclooctat-9-en-7-ol was conducted as previously described.[7] In brief a pET Vector harbouring *cotB2* (GenBank: BAI44338.1) or its mutants and a pACYC vector harbouring polycistronic genes of *dxs*-pathway[8] were transformed to *E. coli* HMS174(DE3) (Merk Millipore). Cultivation was carried out in BasalR-Media with 3 % glycerol as carbon source. A pre-culture was diluted to an OD of 0.1 and protein expression induced with 0.5 mM IPTG. After cultivation at 28° C for three days, the products were extracted with ethyl acetate and analysed by GC/MS.

Analytics of diterpenes. Diterpenes as products of the CotB2 enzyme and its variants were analysed by a Trace GC Ultra with DSQII (Thermo Scientific). 1 µl sample was loaded by a TriPlus auto-sampler onto a SGE BPX5 column (30 m, I.D 0.25 mm, film 0.25 µm). Initial column temperature was set to 50 °C and maintained for 2.5 min before a temperature gradient at 10 °C*min^{-1} up to 320 °C was applied. The final temperature was kept for additional 3 min. MS data were recorded at 70 eV (EI) and m/z (rel. intensity in %) as total ion current (TIC). The recorded m/z range was in between 50 to 650.

Supplementary Table 1. CotB2^{wt} and CotB2 variants as well as the altered products. The numbering of compounds is according to Supplementary Figure 2.

Variant	Product	Compound	Reference
wild-type	cyclooctat-9-en-7-ol	1	[9]
V80L	no product	-	this study
N103A	3,7,12-dolabellatriene	2	[3]
F107A	*R*-Cembrene A	3	[7]
F107Y	cyclooctat-1,7-diene	5	[7]
F107L	cyclooctat-9-en-7-ol<br>3,7-Dolabelladiene-9-ol<br>cyclooctat-6-en-8-ol	1<br>5<br>6	[7]<br>[3]
F149L	cyclooctat-7-en-3-ol	7	[7]
V150A	cyclooctat-9-en-7-ol	1	this study
F185A	cyclooctat-9-en-7-ol<br>cyclooctat-6-en-8-ol	1<br>6	[3]
W186L	cyclooctat-9-en-7-ol<br>cembrane A,<br>3,7,18-dolabellatriene	1<br>3<br>8	[3]
W186F	cyclooctat-9-en-7-ol<br>cyclooctat-6-en-8-ol<br>3,7-dolabelladiene-9-ol<br>cyclooctat-7-en-3-ol	1<br>6<br>5<br>7	[3]
W186H	3,7,18-dolabellatriene<br>cyclooctat-7-en-3-ol	8<br>7	[3]
M189C	cyclooctat-9-en-7-ol	1	this study
L281V	cyclooctat-9-en-7 ol	1	this study
W288F	cyclooctat-9-en-7-ol	1	this study
W288G	3,7,18-dolabellatriene	8	[10]

Supplementary Table 2. Crystallographic data collection and refinement statistics.

	CotB2^{wt}•Mg^{2+}_3•F-Dola	CotB2^{wt}•Mg^{2+}_3•AHD	CotB2$^{\Delta C}$•Mg^{2+}_B	CotB2^{F107A}•Mg^{2+}_B
**Data collection**				
Space group	$P2_12_12_1$	$P1$	$P2_12_12_1$	$P2_12_12_1$
Cell dimensions				
a, *b*, *c* (Å)	62.9 98.7 105.9	53.4 56.5 57.0	61.0 100.0 107.7	61.4 100.1 107.9
α, β, γ (°)	90.0 90.0 90.0	91.8 101.7 115.9	90.0 90.0 90.0	90.0 90.0 90.0
Resolution (Å)	44.74 - 1.80 (1.87 - 1.80)	20.00 - 2.10 (2.18 - 2.10)	50.00 - 2.15 (2.23 - 2.15)	47.50 – 1.90 (1.95 – 1.90)
R_{meas} (%)	11.4 (114.6)	14.0 (92.2)	33.3 (208.7)	12.2 (164.6)
$I / \sigma I$	14.4 (1.9)	7.6 (1.4)	6.2 (1.0)	12.2 (1.4)
Completeness (%)	99.6 (98.8)	99.1 (99.1)	99.8 (99.0)	99.9 (99.9)
Redundancy	6.6 (6.7)	3.1 (2.8)	6.6 (6.7)	7.3 (7.4)
**Refinement**				
Resolution (Å)	44.74 – 1.80	19.83 – 2.10	49.86 – 2.15	47.50 – 1.90
No. reflections	61142	33590	36480	53118
R_{work} / R_{free}	0.17 / 0.20	0.18 / 0.24	0.20 / 0.24	0.28 / 0.21
No. atoms				
Protein	2439 (A), 2484 (B)	2320 (A), 2438 (B)	2273 (A), 2271 (B)	2309 (A), 2297 (B)
Ligand/ion	21 (EXW), 9 (PPV)	14 (AHD)	-	-
Water	283	223	197	246
B-factors				
Protein	24.4 (A), 25.8 (B)	36.3 (A), 33.7 (B)	32.9 (A), 33.4 (B)	32.2 (A), 32.7 (B)
Ligand/ion	28.0 (EXW), 17.8 (PPV)	30.90 (AHD)	-	-

Water	31.1	33.0	34.4	37.3
R.m.s. deviations				
Bond lengths (Å)	0.012	0.007	0.007	0.007
Bond angles (°)	1.291	0.845	0.806	0.776

*Values in parentheses are for highest-resolution shell.

Supplementary Table 3. Results of a DALI search[11] with the structure of CotB2^{wt}•Mg^{2+}_3•F-Dola.

PDB ID	rmsd [Å]	sequence identity [%]	Z-score	protein	TPS family	Ligand in active site	resolution [Å]	Lit.
5gue	0.8	100	46.5	cyclooctat-9-en-7-ol synthase (CotB2)	diterpene	GGSDP, 1 Mg^{2+}	1.80	[12]
2oa6	3.4	15	20.3	aristolochene synthase	sesquiterpene	PP_i, 3 Mg^{2+}	2.15	[13]
3kb9	3.2	11	18.8	epi-isozizaene synthase	sesquiterpene	PP_i, 3 Mg^{2+}, N-benzyl-N,N-diethylethanaminium	1.60	[2]
4okm	3.4	15	18.4	selinadiene synthase	sesquiterpene	PP_i, 3 Mg^{2+}	2.10	[14]
5a0i	3.4	11	18.0	labdane-related diterpene synthase (LrdC)	diterpene	PP_i, 2 Mg^{2+}	2.57	[15]
5dz2	3.3	12	17.8	germacradienol/ geosmin synthase	sesquiterpene	alendronate, 3 Mg^{2+}	2.11	[16]
5erm	3.0	17	17.2	fusicoccadiene synthase	diterpene	pamidronate, 3 Mg^{2+}	2.30	[17]
1jfa	3.8	13	17.1	trichodiene synthase	sesquiterpene	no ligand	2.50	[18]
4la6	3.6	10	16.0	2-methylisoborneol synthase	monoterpene	2-fluorolinalyl diphosphate, 2 Mg^{2+}	2.00	[19]
4xlx	3.6	10	15.7	*ent*-kaurene synthase (BjKS)	diterpene	no ligand	2.00	[20]

Supplementary Table 4. Root-mean-square deviation (rmsd) given in Å for superposition of different CotB2 structures.

	$CotB2^{wt}$	$CotB2^{wt}$• Mg^{2+}_{3}•F-Dola	$CotB2^{F107A}$• Mg^{2+}_{B}	$CotB2^{wt}$• Mg^{2+}_{B}•GGSDP	$CotB2^{\Delta C}$
$CotB2^{wt}$ (open)	-	0.7	0.5	0.3	0.4
$CotB2^{wt}$• Mg^{2+}_{3}•F-Dola (closed)	0.7	-	0.4	0.7	0.5
$CotB2^{F107A}$• Mg^{2+}_{B}	0.5	0.4	-	0.5	0.2
$CotB2^{wt}$• Mg^{2+}_{B}•GGSDP	0.3	0.7	0.5	-	0.4
$CotB2^{\Delta C}$	0.4	0.5	0.2	0.4	-

Supplementary Table 5. Oligonucleotides used for cloning.

**number**	**description**	**oligonucleotide sequence (5' → 3')**
1	fw Cter deletion	TATACCATGGCAGGCGCACAGGATATTG
2	rv Cter deletion	TATAAAGCTTTTAACGTTTGTTGGAGGTGGTC
3	fw V80L	GATTAGCTATGTTGGTTTAGTTCTGTGGTC
4	rv V80L	CAACATAGCTAATCCAACGTTCATCACTAAC
5	fw V150A	CAGCACGTGCATTTGCTACCAGCGATCAC
6	rv V150A	CAAATGCACGTGCTGCTTCATAGGCAACTTC
7	fw M189C	GATTTTTGGATGAAATGTAGCTATCCGATTTATC
8	rv M189C	CAAAAATCCACGCCAATATCGGTAAC
9	fw L281V	GATGTTTTTCTGGATGTGATTTATGGCAATTTTG
10	rv L281V	CAGAAAAACATCCTGTGTCAGCTGATC
11	fw W288F	CACCTCCAACAAACGTTATAAAAC
12	rv W288F	GTTGGAGGTGGTAAACACAAAATTG

Supplementary Table 6. Total and interaction energies[a] (kcal/mol) between CotB2 active site amino acid moieties and carbocation intermediates.

	systems	total energies (Hartree)	counterpoise corrected total energies (Hartree)	complexation energies (kcal/mol)
**A**	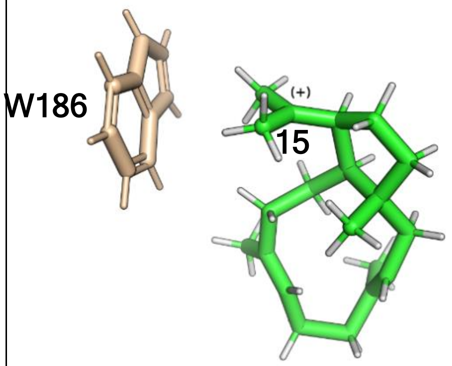	-1145.025566110	-1145.043565256	-11.3
**B**	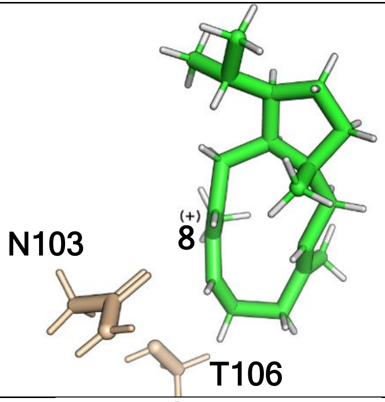	-1106.162596787	-1106.191610934	-18.2
**C**	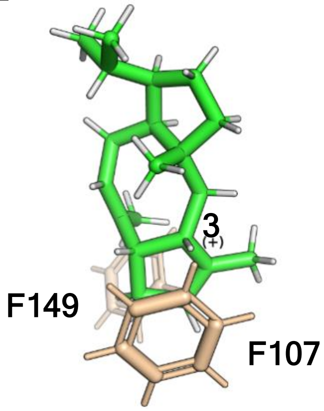	-1245.649659045	-1245.665518468	-10.0

**E**	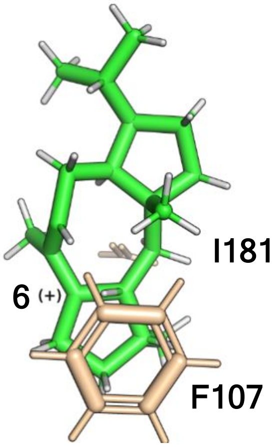	-1127.952930905	-1127.978105188	-15.8
**G**	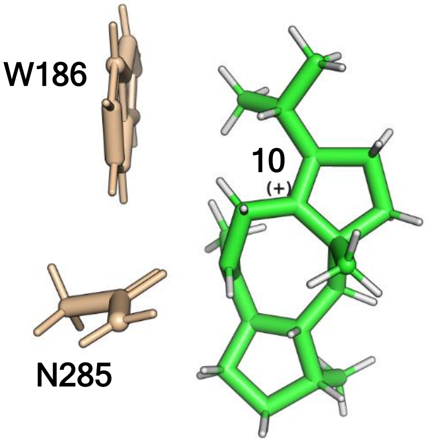	-1354.174380348	-1354.205374990	-19.4
**H**	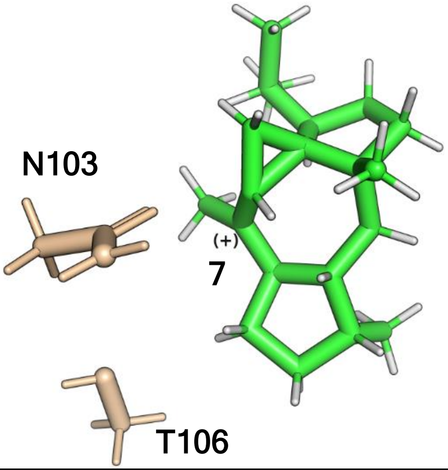	-1106.197299196	-1106.217665068	-12.8

I	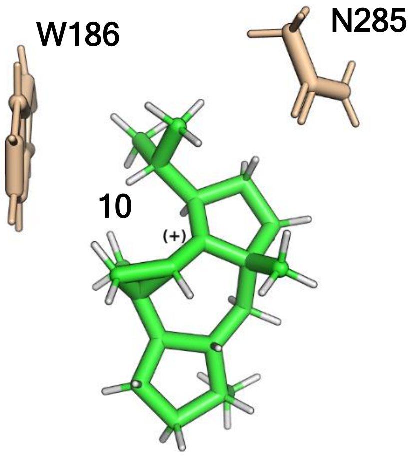	-1354.204624524	-1354.229503270	-15.6

[a] The interaction energies were computed as the energy of the complex relative to the energy of the individual molecular fragments. The structures were taken from the QM(SCCDFTB)/MM simulations after energy minimization, as described in the Methods section. The interaction energies were obtained from M06-2X/6-31+G(d,p) single point calculations, and include counter-poise correction to remove basis-set superposition error.

Supplementary Table 7. Cartesian coordinates of complexes presented in Supplementary Table 6.

intermediate A

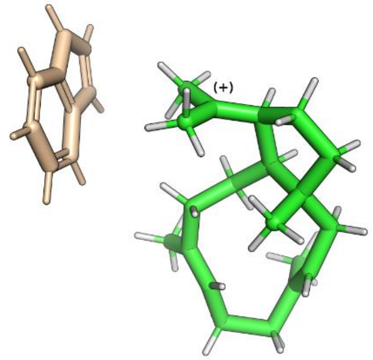

atom	x	y	z
C	5.229	-1.115	-4.849
C	5.743	0.050	-5.367
H	6.606	0.572	-4.968
N	5.051	0.385	-6.507
H	5.313	1.017	-7.223
C	4.082	-0.539	-6.739
C	4.147	-1.503	-5.707
C	3.237	-2.558	-5.694
H	3.241	-3.298	-4.908
C	2.309	-2.651	-6.733
H	1.612	-3.478	-6.745
C	3.158	-0.628	-7.763
H	3.148	0.096	-8.562
C	2.274	-1.706	-7.753
H	1.555	-1.813	-8.552
H	5.576	-1.641	-3.960
C	-1.895	-1.089	-1.862
H	-1.713	-0.654	-2.840
H	-2.963	-1.034	-1.666
H	-1.623	-2.137	-1.906
C	2.537	-4.315	-1.353
H	3.072	-4.584	-2.265
H	2.489	-5.203	-0.733
H	3.130	-3.558	-0.833
C	0.377	-3.144	2.063
H	0.285	-3.677	3.010
H	0.497	-2.085	2.278
H	1.291	-3.506	1.586
C	-1.434	-0.977	0.661
H	-0.660	-0.664	1.367
H	-2.377	-0.537	1.009
C	-1.590	-2.469	0.628
H	-2.441	-2.822	0.046
C	-0.816	-3.417	1.201
C	-1.103	-4.877	0.899
H	-2.145	-4.976	0.582
H	-0.957	-5.496	1.792
C	-0.191	-5.414	-0.244
H	-0.656	-6.299	-0.685
H	0.766	-5.728	0.172
C	0.008	-4.340	-1.272
H	-0.916	-3.856	-1.591
C	1.166	-3.796	-1.685
C	1.134	-2.417	-2.325
H	0.197	-2.259	-2.854
H	1.939	-2.288	-3.058
C	1.306	-1.421	-1.145
H	2.341	-1.067	-1.140
H	1.187	-1.983	-0.210

intermediate B

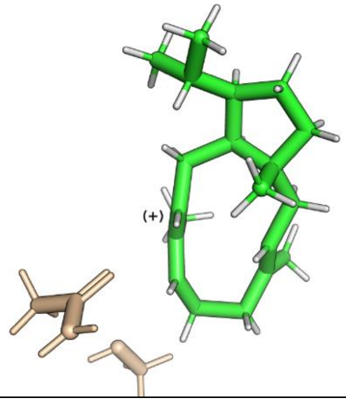

atom	x	y	z
C	-2.108	-0.451	-1.760
H	-2.065	0.227	-2.606
H	-3.082	-0.340	-1.291
H	-2.033	-1.468	-2.139
C	2.018	-3.588	-0.640
H	2.784	-3.008	-1.150
H	2.418	-4.578	-0.438
H	1.819	-3.103	0.314
C	-0.011	-3.243	2.384
H	-0.562	-3.558	3.270
H	0.288	-2.209	2.519
H	0.889	-3.861	2.330
C	-1.092	-0.964	0.546
H	-0.209	-0.773	1.161
H	-1.945	-0.582	1.124
C	-1.301	-2.435	0.359
H	-1.958	-2.724	-0.459
C	-0.867	-3.431	1.169
C	-1.241	-4.849	0.837
H	-2.220	-4.892	0.358
H	-1.267	-5.470	1.735
C	-0.206	-5.563	-0.147
H	-0.617	-6.540	-0.379
H	0.752	-5.670	0.367
C	-0.124	-4.745	-1.343
H	-0.957	-4.804	-2.046
C	0.763	-3.671	-1.463
C	0.288	-2.590	-2.203
H	-0.639	-2.748	-2.754
H	0.209	0.490	-4.053
C	0.923	-1.284	-2.316
H	0.773	-0.988	-3.359
H	2.000	-1.380	-2.140
C	0.436	-0.088	-1.392
H	1.131	-0.110	-0.542
C	-0.998	-0.107	-0.756
C	-1.152	1.388	-0.363
H	-0.566	1.590	0.537
H	-2.189	1.645	-0.141
C	-0.596	2.163	-1.548
H	-0.279	3.171	-1.270
H	-1.372	2.271	-2.305
C	0.594	1.331	-2.073
H	1.515	1.768	-1.655
C	0.774	1.335	-3.626
C	2.258	1.158	-3.964
H	2.831	2.009	-3.601
H	2.400	1.091	-5.038
H	2.679	0.265	-3.511

C	0.408	-0.210	-0.951
H	0.770	0.251	-0.018
C	-1.135	-0.335	-0.759
C	-1.564	1.153	-0.812
H	-1.276	1.656	0.116
H	-2.641	1.275	-0.928
C	-0.797	1.745	-1.998
H	-0.682	2.827	-1.907
H	-1.327	1.545	-2.932
C	0.600	1.086	-1.999
H	1.325	1.713	-1.458
C	1.201	0.684	-3.251
C	2.672	0.620	-3.337
H	3.167	1.146	-2.525
H	3.029	0.990	-4.299
H	2.979	-0.432	-3.293
C	0.447	0.292	-4.448
H	-0.510	-0.168	-4.208
H	1.023	-0.363	-5.099
H	0.219	1.193	-5.034

C	0.229	2.568	-4.342
H	-0.849	2.643	-4.244
H	0.457	2.498	-5.400
H	0.677	3.483	-3.972
C	-0.028	-6.523	-5.529
H	-0.371	-7.334	-6.171
H	0.366	-6.937	-4.601
C	-1.198	-5.590	-5.215
O	-1.056	-4.597	-4.504
N	-2.377	-5.899	-5.802
H	-3.158	-5.278	-5.688
H	-2.451	-6.711	-6.371
H	0.757	-5.964	-6.039
C	1.137	-8.889	0.094
H	0.968	-8.161	0.888
O	1.171	-8.224	-1.159
H	1.323	-8.908	-1.836
H	2.087	-9.396	0.263
H	0.330	-9.621	0.096

intermediate C

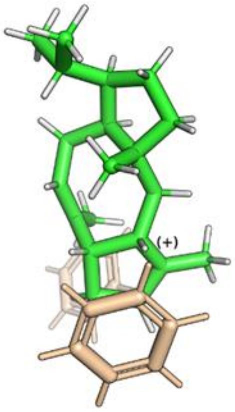

atom	x	y	z
C	-1.357	-0.620	-2.309
H	-0.929	-0.142	-3.185
H	-2.377	-0.255	-2.195
H	-1.418	-1.687	-2.506
C	1.578	-3.587	0.203
H	2.546	-3.108	0.118
H	1.730	-4.661	0.336
H	1.085	-3.225	1.113
C	-1.874	-2.640	2.499
H	-1.841	-3.308	3.352
H	-2.865	-2.160	2.476
H	-1.182	-1.810	2.675
C	-0.855	-1.221	0.156
H	0.036	-1.293	0.788
H	-1.613	-0.719	0.767
C	-1.427	-2.670	-0.051
H	-2.424	-2.551	-0.511
C	-1.606	-3.328	1.239
C	-1.435	-4.790	1.161
H	-2.376	-5.279	1.447
H	-0.715	-5.123	1.923
C	-0.989	-5.116	-0.267
H	-1.771	-5.645	-0.805
H	-0.115	-5.766	-0.269
C	-0.688	-3.777	-0.961
H	-1.119	-3.753	-1.967
C	0.739	-3.321	-1.002

intermediate E

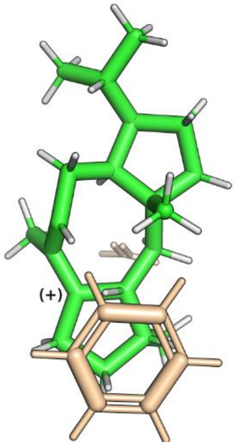

atom	x	y	z
C	-4.331	-5.963	-1.091
C	-4.893	-5.116	-0.118
H	-5.300	-5.538	0.792
C	-4.963	-3.735	-0.329
H	-5.395	-3.090	0.426
C	-4.500	-3.193	-1.532
H	-4.588	-2.132	-1.708
C	-3.854	-5.404	-2.286
H	-3.446	-6.045	-3.054
C	-3.942	-4.026	-2.510
H	-3.596	-3.614	-3.447
H	-4.267	-7.037	-0.919
C	3.224	-1.267	2.636
O	2.554	-1.372	1.612
H	2.798	-0.791	3.519
H	4.244	-1.650	2.661
C	-2.230	-0.604	-1.758
H	-2.426	0.112	-2.548
H	-3.007	-0.483	-1.006
H	-2.326	-1.599	-2.179
C	2.125	-3.077	-1.525
H	2.580	-2.552	-2.359
H	2.598	-4.057	-1.434
H	2.339	-2.528	-0.609
C	-0.610	-3.030	2.669
H	0.458	-3.177	2.525
H	-0.934	-3.639	3.508

C	1.045	-2.435	-1.986
H	0.377	-2.406	-2.845
H	1.173	0.164	-3.888
C	1.924	-1.250	-1.827
H	2.386	-0.974	-2.774
H	2.729	-1.431	-1.111
C	1.024	-0.074	-1.274
H	1.400	0.114	-0.258
C	-0.533	-0.279	-1.051
C	-0.896	1.174	-0.630
H	-0.540	1.356	0.389
H	-1.975	1.349	-0.634
C	-0.156	2.062	-1.623
H	0.080	3.040	-1.192
H	-0.796	2.247	-2.483
C	1.133	1.297	-2.020
H	1.987	1.831	-1.577
C	1.431	1.181	-3.556
C	2.935	1.385	-3.779
H	3.233	2.389	-3.486
H	3.190	1.255	-4.824
H	3.526	0.683	-3.195
C	0.641	2.136	-4.454
H	-0.426	1.940	-4.414
H	0.954	2.004	-5.486
H	0.803	3.178	-4.189
C	-4.445	-5.976	-1.221
C	-4.962	-5.066	-0.279
H	-5.347	-5.432	0.663
C	-5.015	-3.695	-0.564
H	-5.410	-3.004	0.168
C	-4.569	-3.225	-1.805
H	-4.603	-2.169	-2.030
C	-4.009	-5.489	-2.463
H	-3.635	-6.179	-3.205
C	-4.067	-4.122	-2.754
H	-3.729	-3.766	-3.716
H	-4.384	-7.039	-0.990
C	5.253	-7.383	3.291
C	5.069	-6.003	3.464
H	5.933	-5.356	3.509
C	3.781	-5.467	3.606
H	3.650	-4.406	3.762
C	2.663	-6.308	3.564
H	1.673	-5.893	3.689
C	4.120	-8.214	3.220
H	4.251	-9.276	3.078
C	2.832	-7.684	3.369
H	1.971	-8.336	3.339
H	6.256	-7.804	3.213
H	-0.792	-1.986	2.915
C	-0.656	-1.253	0.156
H	0.350	-1.055	0.542
H	-1.343	-0.864	0.915
C	-0.814	-2.765	0.190
H	-2.445	-3.149	1.562
C	-1.395	-3.441	1.420
C	-1.283	-4.955	1.111
H	-2.251	-5.330	0.786
H	-0.990	-5.528	1.989
C	-0.263	-5.107	-0.024
H	-0.553	-5.852	-0.769
H	0.727	-5.403	0.341
C	-0.156	-3.739	-0.612
H	-1.327	-3.306	-0.765
C	0.643	-3.231	-1.748
C	-0.085	-2.627	-2.713
H	-1.136	-2.905	-2.766
H	0.205	0.763	-4.626
C	0.278	-1.329	-3.342
H	-0.490	-1.039	-4.060
H	1.236	-1.351	-3.863
C	0.370	-0.332	-2.142
H	1.245	-0.656	-1.561
C	-0.855	-0.339	-1.125
C	-0.864	1.137	-0.629
H	-0.082	1.277	0.123
H	-1.818	1.409	-0.173
C	-0.538	1.952	-1.873
H	-0.246	2.977	-1.632
H	-1.409	2.009	-2.529
C	0.611	1.170	-2.527
H	1.531	1.472	-2.002
C	0.854	1.425	-4.039
C	2.309	1.102	-4.385
H	2.987	1.797	-3.892
H	2.472	1.175	-5.455
H	2.585	0.100	-4.067
C	0.511	2.849	-4.458
H	-0.541	3.053	-4.288
H	0.706	2.984	-5.516
H	1.106	3.574	-3.908

intermediate G

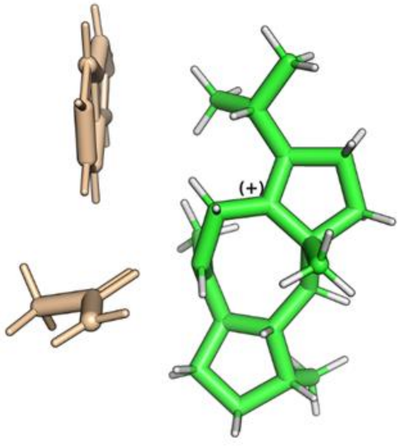

atom	x	y	z
C	-0.048	-6.537	-5.631
H	-0.391	-7.476	-6.066
H	0.439	-6.736	-4.676
C	-1.245	-5.609	-5.410
O	-1.113	-4.486	-4.928
N	-2.434	-6.060	-5.870
H	-3.228	-5.446	-5.829
H	-2.503	-6.967	-6.272
H	0.661	-6.061	-6.309
C	5.154	-1.230	-4.856
C	5.638	-0.141	-5.538
H	6.536	0.405	-5.269
N	4.845	0.098	-6.633
H	5.023	0.717	-7.388
C	3.831	-0.808	-6.669
C	3.980	-1.666	-5.555
C	3.047	-2.678	-5.332
H	3.127	-3.337	-4.480
C	1.984	-2.814	-6.227
H	1.241	-3.584	-6.061
C	2.784	-0.949	-7.561
H	2.694	-0.293	-8.411
C	1.860	-1.965	-7.322
H	1.030	-2.100	-7.995
H	5.580	-1.677	-3.957
C	-2.525	-1.196	-1.626
H	-2.949	-0.542	-2.384
H	-3.209	-1.235	-0.782
H	-2.425	-2.194	-2.046
C	2.357	-2.102	-1.528
H	2.599	-1.739	-2.525
H	3.208	-2.670	-1.154
H	2.245	-1.238	-0.872
C	-0.342	-3.169	2.696
H	0.737	-3.094	2.574
H	-0.538	-3.814	3.549
H	-0.730	-2.178	2.924
C	-0.699	-1.447	0.174
H	0.267	-1.042	0.486
H	-1.430	-1.124	0.921
C	-0.656	-2.980	0.152
H	-2.098	-3.733	1.608
C	-1.010	-3.744	1.453
C	-0.525	-5.184	1.128
H	-1.374	-5.810	0.851
H	-0.067	-5.650	2.004
C	0.475	-5.088	-0.047

intermediate H

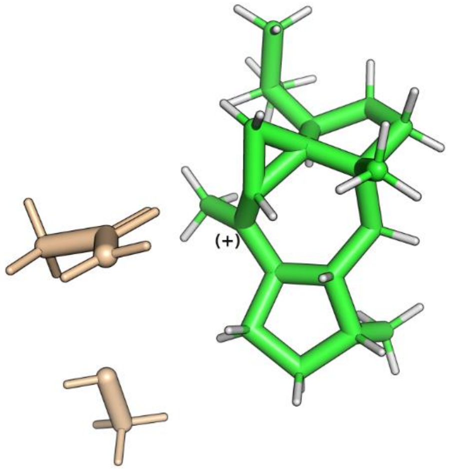

atom	x	y	z
C	-0.044	-6.373	-5.594
H	-0.428	-7.259	-6.099
H	0.434	-6.666	-4.659
C	-1.197	-5.414	-5.299
O	-1.016	-4.338	-4.732
N	-2.406	-5.789	-5.776
H	-3.181	-5.157	-5.688
H	-2.512	-6.663	-6.238
H	0.686	-5.878	-6.235
C	1.161	-8.394	0.040
H	0.999	-7.639	0.809
O	1.131	-7.781	-1.239
H	1.279	-8.491	-1.886
H	2.131	-8.867	0.194
H	0.376	-9.148	0.101
C	-2.752	-0.326	-1.994
H	-2.884	0.465	-2.724
H	-3.571	-0.254	-1.284
H	-2.833	-1.281	-2.507
C	2.062	-2.839	-1.564
H	2.437	-2.598	-2.553
H	2.389	-3.845	-1.295
H	2.525	-2.165	-0.838
C	-1.324	-2.501	2.635
H	-0.235	-2.459	2.624
H	-1.632	-3.047	3.521
H	-1.702	-1.486	2.724
C	-1.420	-0.955	0.112
H	-0.561	-0.621	0.700
H	-2.312	-0.617	0.648
C	-1.436	-2.493	0.071
H	-2.966	-3.192	1.455
C	-1.870	-3.188	1.391
C	-1.333	-4.640	1.247
H	-2.150	-5.344	1.101
H	-0.810	-4.947	2.156
C	-0.381	-4.666	0.041
H	-0.871	-5.086	-0.840
H	0.526	-5.248	0.224
C	-0.062	-3.170	-0.198
H	-2.151	-2.826	-0.694
C	0.584	-2.776	-1.483
C	-0.269	-2.231	-2.446
H	-1.315	-2.508	-2.336
H	2.530	-0.575	-2.782
C	-0.063	-1.146	-3.441
H	-0.899	-1.029	-4.123

H	0.039	-5.506	-0.958
H	1.401	-5.631	0.150
C	0.732	-3.586	-0.209
H	-1.376	-3.337	-0.598
C	1.127	-2.959	-1.521
C	0.207	-2.938	-2.518
H	-0.651	-3.604	-2.460
H	0.273	0.436	-4.718
C	0.033	-1.691	-3.334
H	-0.817	-1.791	-4.024
H	0.894	-1.435	-3.958
C	-0.244	-0.604	-2.359
H	1.463	-3.276	0.548
C	-1.151	-0.659	-1.149
C	-1.289	0.842	-0.725
H	-0.643	1.042	0.134
H	-2.310	1.082	-0.425
C	-0.828	1.656	-1.924
H	-0.441	2.637	-1.649
H	-1.647	1.807	-2.629
C	0.263	0.766	-2.566
H	1.141	0.835	-1.887
C	0.757	1.123	-4.009
C	2.270	0.926	-4.105
H	2.796	1.642	-3.479
H	2.603	1.065	-5.128
H	2.562	-0.073	-3.786
C	0.368	2.539	-4.419
H	-0.710	2.658	-4.438
H	0.740	2.741	-5.417
H	0.792	3.273	-3.740

H	0.904	-1.056	-3.926
C	-0.166	-0.557	-2.077
H	0.632	-2.863	0.601
C	-1.415	-0.167	-1.266
C	-1.142	1.327	-0.938
H	-1.591	1.603	0.019
H	-1.601	1.958	-1.699
C	0.379	1.511	-0.936
H	0.753	1.754	0.057
H	0.661	2.345	-1.581
C	1.014	0.187	-1.441
H	1.346	-0.369	-0.551
C	2.268	0.396	-2.336
C	3.446	0.811	-1.454
H	3.211	1.707	-0.885
H	4.325	1.014	-2.062
H	3.687	0.022	-0.745
C	2.058	1.381	-3.489
H	1.129	1.189	-4.019
H	2.878	1.292	-4.196
H	2.034	2.409	-3.138

intermediate I

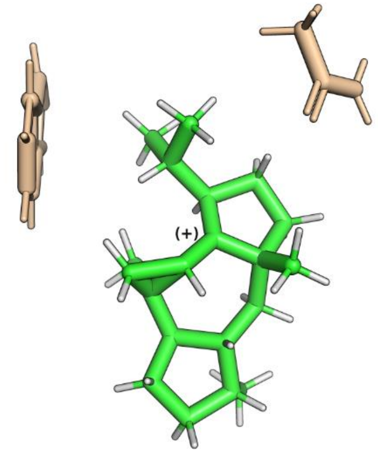

atom	x	y	z
C	5.122	-1.122	-4.851
C	5.613	-0.027	-5.518
H	6.530	0.492	-5.260
N	4.791	0.267	-6.578
H	4.967	0.887	-7.331
C	3.751	-0.610	-6.606
C	3.918	-1.510	-5.529
C	2.979	-2.520	-5.320
H	3.084	-3.220	-4.505
C	1.889	-2.605	-6.189
H	1.143	-3.377	-6.041
C	2.672	-0.695	-7.466
H	2.566	-0.002	-8.285
C	1.735	-1.703	-7.236
H	0.871	-1.786	-7.873

H	5.561	-1.603	-3.977
C	-1.292	5.421	-4.699
H	-0.827	6.377	-4.457
H	-2.122	5.584	-5.386
C	-1.810	4.768	-3.419
O	-2.375	3.674	-3.455
N	-1.652	5.447	-2.262
H	-2.044	5.046	-1.439
H	-1.238	6.365	-2.273
H	-0.556	4.768	-5.168
C	-2.695	0.396	-0.789
H	-2.861	1.163	-1.542
H	-3.212	0.685	0.120
H	-3.141	-0.533	-1.137
C	1.867	-2.085	-1.562
H	2.221	-1.581	-2.459
H	2.452	-3.001	-1.454
H	2.086	-1.472	-0.691
C	-0.486	-2.916	2.753
H	0.547	-2.969	2.413
H	-0.616	-3.655	3.540
H	-0.655	-1.929	3.179
C	-0.943	-0.792	0.677
H	0.091	-0.688	1.017
H	-1.580	-0.464	1.504
C	-1.240	-2.272	0.386
H	-2.488	-3.025	2.007
C	-1.472	-3.184	1.621
C	-1.333	-4.621	1.044
H	-2.307	-5.105	0.983
H	-0.716	-5.243	1.697
C	-0.698	-4.497	-0.354
H	-1.455	-4.597	-1.136
H	0.064	-5.257	-0.538
C	-0.112	-3.078	-0.352
H	-2.165	-2.342	-0.203
C	0.417	-2.487	-1.646
C	-0.788	-1.444	-2.272
H	-1.750	-1.786	-1.899
H	1.169	-0.251	-3.886
C	-0.133	-2.563	-3.019
H	-0.845	-3.349	-3.238
H	0.561	-2.336	-3.821
C	-0.412	-0.215	-1.726
H	0.745	-3.081	0.336
C	-1.188	0.243	-0.506
C	-0.554	1.622	-0.149
H	0.104	1.513	0.714
H	-1.313	2.360	0.112
C	0.253	2.038	-1.376
H	1.100	2.677	-1.124
H	-0.378	2.582	-2.079
C	0.724	0.709	-2.004
H	1.561	0.358	-1.369
C	1.250	0.765	-3.471
C	2.728	1.160	-3.479
H	2.859	2.164	-3.086
H	3.116	1.144	-4.492
H	3.322	0.476	-2.875
C	0.432	1.704	-4.358
H	-0.633	1.551	-4.217
H	0.673	1.537	-5.403
H	0.651	2.745	-4.130

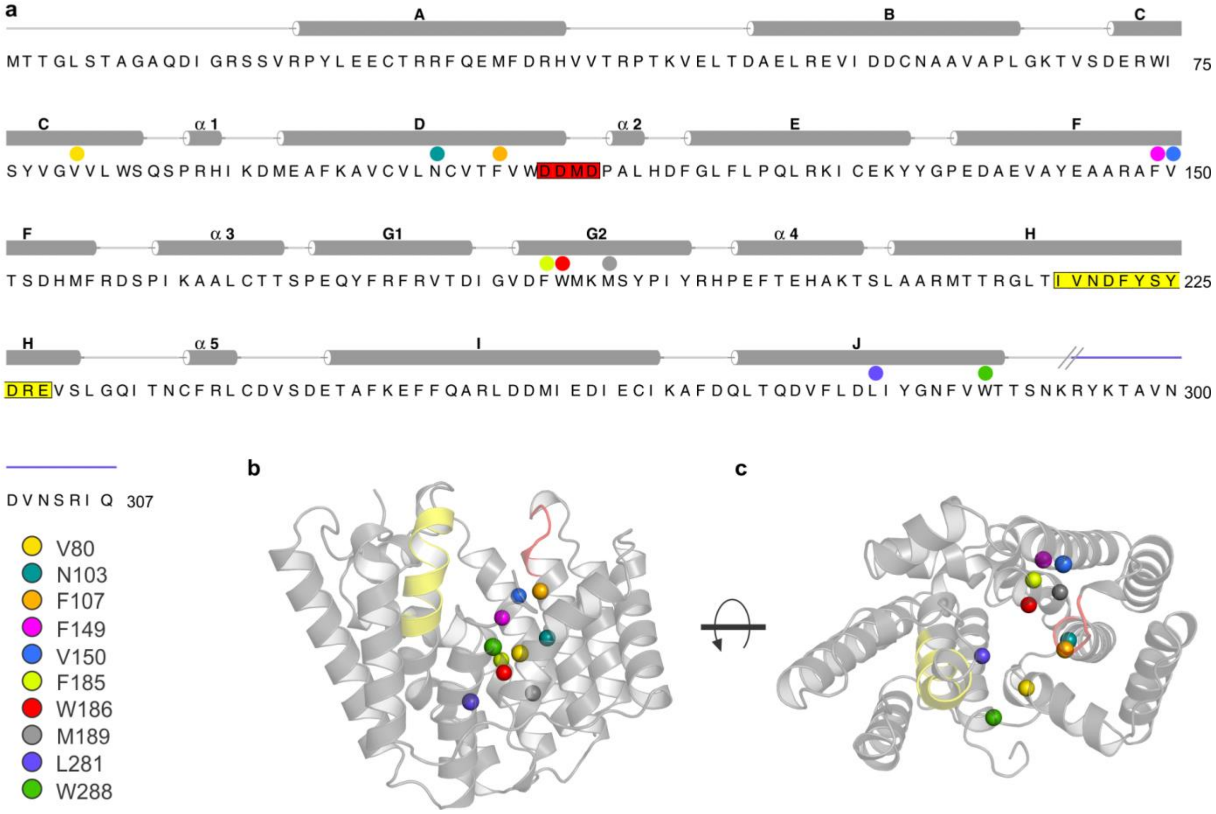

Supplementary Figure 1. Secondary structure elements and variants of CotB2. a, On top of the primary sequence of CotB2 the secondary structure elements are drawn. Slashed lines at the C-terminus indicate the terminal residue of the open state of CotB2. Missing residues have not been built due to their flexibility. The aspartate-rich motif (^{110}DDXD^{113}) is highlighted in red and the ^{218}NSE^{228} motif in yellow. CotB2 variants, as listed in Supplementary Table 1 are indicated by circles. For colour-coding see inlet. The structured C-terminus of CotB2 in the closed conformation is shown in purple. **b,** The open structure of CotB2^{wt} (PDB-ID 4OMG) is shown in gray cartoon representation. The ^{110}DDXD^{113} motif is shown in red and the ^{218}NSE^{228} motif in yellow. CotB2 variants are indicated by colored spheres. Color-coding of variants as in (a). **c,** View into the hydrophobic pocket of CotB2^{wt}. View rotated by 90 ° in respect to (b).

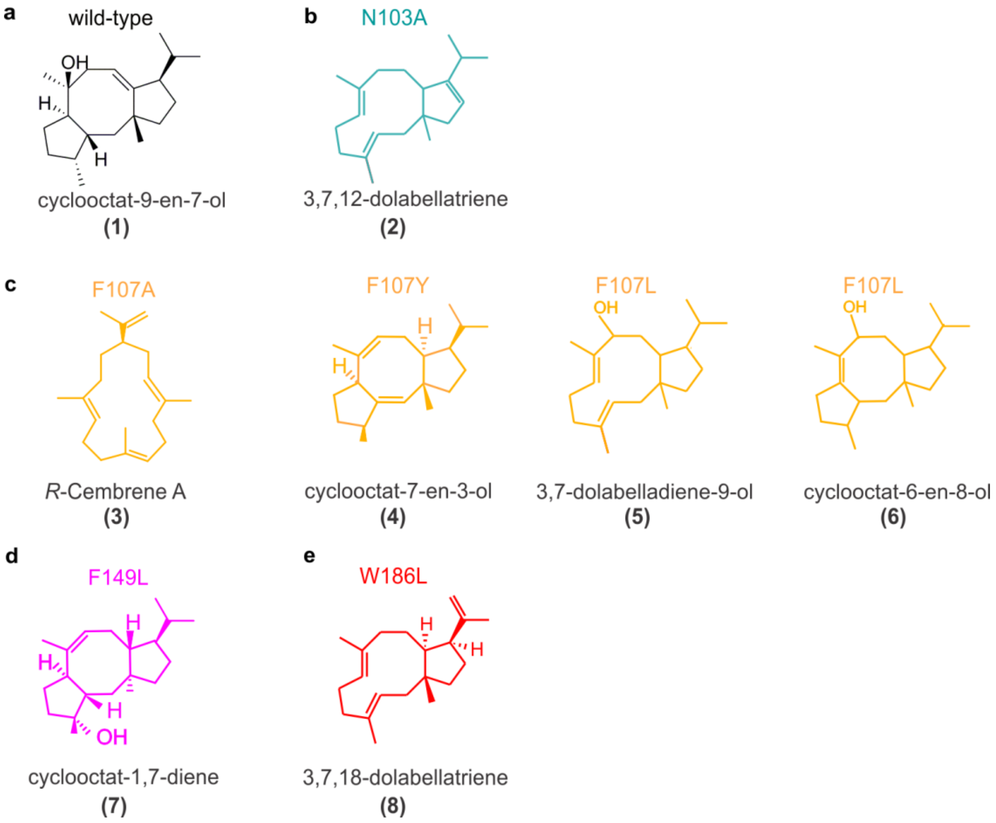

Supplementary Figure 2. Overview of products of CotB2^{wt} and its variants. Some colour coding of variants and their products as in Supplementary Table 1 and Supplementary Figure 1. **a,** cyclooctat-9-en-7-ol (1) produced by wild-type CotB2. **b,** Variant N103A producing 3,7,12-dolabellatriene (2) **c,** Variant F107A producing *R*-Cembrene A (3), F107Y producing cyclooctat-1,7,-diene (4), and F107L producing a product mixture composed of cyclooctat-9-en-7-ol (1), 3,7-dolabelladiene-9-ol (5), and cyclooctat-6-en-8-ol (6). **d,** Variant F149L producing cyclooctat-6-en-8-ol (7). **e,** Variant W186L producing cyclooctat-9-en-7-ol (2) and *R*-Cembrene A (3) in addition to 3,7,18-dolabellatriene (8).

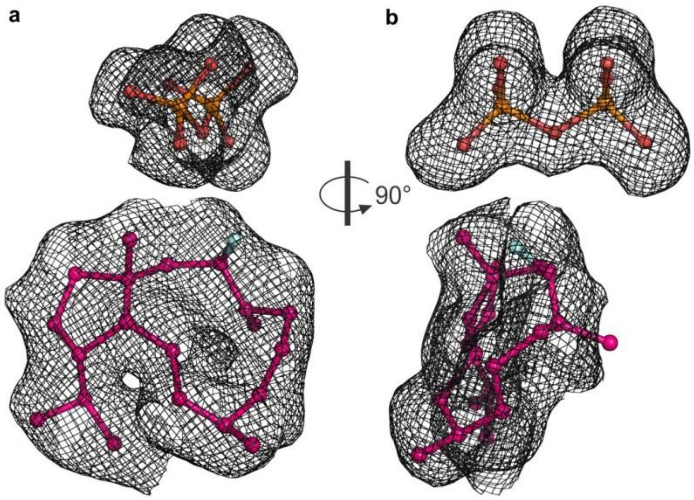

Supplementary Figure 3. Polder electron density maps[21] shown as mesh at a σ-level of 2.0. Molecules are presented as ball-stick-models with carbon atoms colored in magenta, oxygen in red, phosphorous in orange and fluorine in light blue. **a,** Structure of CotB2^{wt}•Mg^{2+}_{3}•F-Dola with a polder electron density map around the F-Dola and diphosphate molecule. **b**, view in a, rotated by 90°. The polder electron density of the diphosphate and intermediate is clearly not connected, arguing for a cleaved diphosphate moiety.

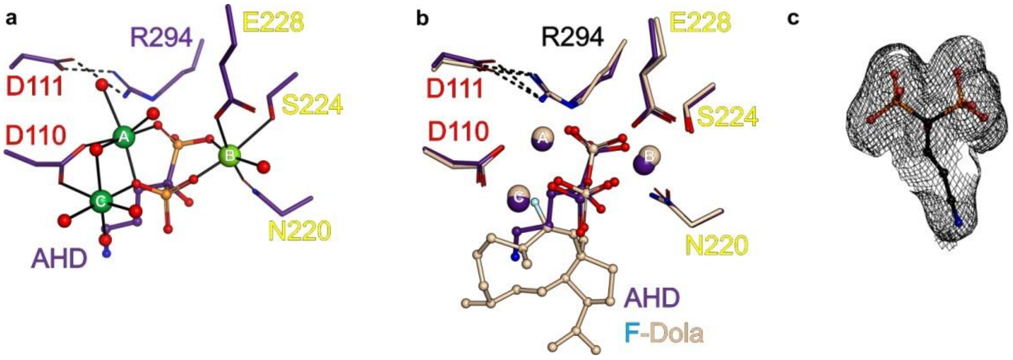

Supplementary Figure 4. a, In the structure of CotB2^{wt}•Mg^{2+}_3•AHD, the bisphosphonate moiety of alendronate (AHD) is coordinated by three Mg^{2+}-ions. The strong binding of AHD to the active site is reflected in low B-factors of 24 Å^2. The entire C-terminus is observed and folds over the active site. Residues of the DDXD motif are labelled with red letters, residues of the NSE motif with yellow letters respectively. **b,** Superposition of CotB2^{wt}•Mg^{2+}_3•AHD and CotB2^{wt}•Mg^{2+}_3•F-Dola. The coordination of the Mg^{2+} ions is identical in both structures. **c,** Structure of CotB2^{wt}•Mg^{2+}_3•AHD with a polder electron density map around AHD molecule shown as mesh at a σ-level of 2.0. The AHD molecule is presented as ball-stick-model with carbon atoms colored in black, oxygen in red, phosphorous in orange and nitrogen in blue.

> cyclooctat-9-en-7-ol synthase (WP_093468823), *Streptomyces melanosporofaciens*, PDB-ID 6GGI
MTTGLSTAGAQDIGRSSVRPYLEECTRRFQEMFDRHVVTRPTKVELTDAELREVIDDCNAAVAPLGKTVSDERWISY
VGVVLWSQSPRHIKDMEAFKAVCVLNCVTFVW**DDMD**PALHDFGLFLPQLRKICEKYYGPEDAEVAYEAARAFVTSDH
MFRDSPIKAALCTTSPEQYFRFRVTDIGVDFWMKMSYPIYRHPEFTEHAKTSLAARMTTRGLTIV**NDFYSYDRE**VSL
GQITNCFRLCDVSDETAFKEFFQARLDDMIEDIECIKAFDQLTQDVFLDLIYGNFV**W**TTSNK**RY**KTAVNDVNSRIQ

>labdane-related diterpene synthase (WP_019525557), *Streptomyces*, PDB-ID: 5A0K
MTDTDDGGTMLPLPDFTATFPEPFPAGPHSERTEHRLLDWLEEHPLLPSAKAKAVLVNITSHGASRTFPTADADDLL
LFAELLLWLTAF**DDVHAE**GNGVGGPAALVDRASELMLVLAGGNPPRAMSPFPAVLHDLLARFRARASAAAYHRLAAS
LRDTLMALVWEAHHVAKPEGVALATYLAMRPHTVFIKTITAAGEILLGYELTDTQRALAAVRNLETAVANLAGWI**ND**
LASYEREMQRGRGQPLSLPTLLHARHGGTIEEAFTRASSMCENEAAVARRGITHLAHASPNALTAHARALEDITRSF
I**W**HTSHA**RY**QGIRPNRGSSTSSPAR

> iso-elsabellatriene synthase(WP_003963279), *Streptomyces clavuligerus*
MTISVPQLDCPLSRPVHPEGERADAYAVEWLRGVGLMADEADAAPVLAVGLGRLAACYVDENASWDTLAFMTILLAW
YAEY**DDRAID**STGAIDGLTDAEVAELHRALGEILRDRPAPDPSDPVQRGLADVWRTLNGLASDWDRAAFVDTTLRYF
EANRYERVNIRRGIPPTPSAHIGMRRHGGHVYGMYILGAAVNGYRPERRVLDHAAVRELETLAANYTSWA**NDLHSFA**
REHRMGQVNNLVWSVHHHEGLTFQQAADRVADLCDKELAAYLELRQTLPELGIPLTGATGRHVRFLEDMMWSMVD**W**S
ARSA**RY**DVVPEAA

> terpene cyclase (WP_046708564.1), *Streptomyces europaeiscabiei*
MGDAGLAHIPEIHCPFPYRVNPHADRARAHLADWVQRTGLVHRESARKRFDQADFGWFAALVYPTASLRHLELMADW
FAWLFLV**DDQLD**DGSAGRSPDRMQQMVAGMHDVLGSPDFGVSLLRDPDVPPAVASLAELWQRTAVDAPAHWRRRFIR
HLRDCLVTATVWEGGNRIRGIVPDEVTYIENRRHTGAIYVCMDLIDIVERLDVPEELYDSAEFTRALDAACNVVCWT
NDVYSLDKERSLGEVHNIAYLAQYHRGLDREQALAEVCAATSAETERFLAAERHLLSARPGQSAVLAPYLAGMRTWI
RGNLD**W**SRRTK**RY**QAGTAVSWARPADYVERALIGVDQ

> diterpene synthase(BAP82252), *Streptomyces avermitilis*
MNVIDFPQVDLGTEATISPDVDKAGEHLKTWSRATGIVLDGPNGDAFDALAQHRLAAWTYPAATGAELDLMADWIGW
LFAF**DDVFD**ESIDGCDQDFAIATAAATNTVYTGLPSAPPSPVVRPYVVALEDLWERTTQGMPAYWCRRLANDMVDYV
NSYRSHALINASRIALDEQSYRAHRLISSAVFITLDLGEAAARRALSESLLVHPYIRAAREAANNIVSWS**NDLYSAP**
KELSLGDLCNYIAVLQSQDDLTTAQAADRVSQYLHEEIARFDQTKQLIHTHLIPRLPTEDQQGVISMLDTCSNWITG
NVA**W**SLETA**RY**ASRAIDMEREKEVFGDLRT

> diterpene synthase (BAP82229), Streptomyces sp. ND90
MPFVPDFTTPFRYRLNPHLAEVTPRARQWMLESDLVDDTHMLEYEMARIPELMAAAYPGASADDLLLSCDLMGIMFA
I**EDED**CGSHPRHSVAGIATRCKAMIQVMGGVDPGADDPVVLAFSDTWHRLCDGMSDTWVVRHRSSWKDFLDNHHTWE
PVVVEKRGMPTLEDYLWERAYSSGMYVLYDWSERFSADRAEIPQPALEDPRLATLHRNCIYTIIAI**NDTHSLERE**IR
RNDPVPNLLKVLMHHERLTVEQSVERAKKMLADAIEEYLEVEFEYLAHWWRSGLPPRQMGAVEQRLRDMRNWTSSNC
R**W**HCIVP**RY**DHVARDPERDRPGLPTQPAGRQGKDT

> Hydropyrene synthase (EFG04252), *Streptomyces clavuligerus* ATCC 27064
MTISVPQLDCPLSRPVHPEGERADAYAVEWLRGVGLMADEADAAPVLAVGLGRLAACYVDENASWDTLAFMTILMAW
YAEY**DDRAID**STGAIDGLTDAEVAELHRALGEILRDRPAPDPSDPVQRGLADVWRTLNGLASDWDRAAFVDTTLRYF
EANRYERVNIRRGIPPTPSAHIGMRRHGGHVYGMYILGAAVNGYRPERRVLDHAAVRELETLAANYTSWA**NDLHSFA**
REHRMGQVNNLVWSVHHHEGLTFQQAADRVADLCDKELAAYLELRQTLPELGIPLTGATGRHVRFLEDMM**W**SMVDWS
ARSA**RY**DVVPEAA

> terpene cyclase (WP_084744884.1), *Kitasatospora albolonga*
MDSELPDIYCPFPQRTNPHVGHTRGHLATWIRQTGLVHRESAMHRFEQADFGAFVGMVYPTAGPEHLDLVADWFVWL
FLV**DDQLD**DGHLGRSPELVRAVVERMRAVVDGSAPAPLPGEELPAAVIALMDLWERTTPNAAPHWRTRFAWHLVTYL
TTATTWEAGNRADDVVPSEETYIAKRRHTGAIHVCMDLIEIVTGIEAPESLHNDPRFITALEAACNHVCWA**NDVYSF**

EKEQVLGEIHNLVHLVRHHRGLGEQQALDHVAERLALETERFLTAEDELLELYPELTGLLVPYLEGMRSWMRGNLD**W**
SRQTP**RY**NPADVGQYEEPQQYLEETVLGIAPDHAEAAPCAAEAPRQG

> tsukubadiene synthase (EIF90392), *Streptomyces tsukubaensis* NRRL 18488
MIEVPPFWCPLPIAIHPAADQAEKDARAWAERYGVRLRIADQVQPGRLGAYWAPHGTYEGMLAVGCWNFWAFAF**DDH**
LDEPLPLDVPVTTSLVQQAVDIPSPPITDDPWAAGAQAVFNMFRDLATPTQVRYCADNHRRWLHGACWRHSNHVNRR
LPPLAEYIPLRMQDAAAQATCLIAVLIGSDISVPEQEMDSPRVRALLETASWTATID**SDLHSFQLE**DTQRPVSQHIV
SVLMHERGIGVDEALRQSVALRDRFMTRFLHLQQECARTGSSELARFAHTLGYVISGYLQ**W**AVDTS**RY**GQTEATFSF
TDTPRDDTPEPPPGIPSVEWLWTL

> terpene synthase(ZP_00085244), *Pseudomonas fluorescens* PfO-1
MRKFRQRFYMDLSSLHRNRVGEHLNRPREKDHPMNQSSSARTPRSATAPFIVRAVRCPPPTRIDEALGQEVNERLME
WISNIGIFAGKEEKIRASDFGRYAMLCHADTNNPDRLLLVAQCFAALFAV**DDHYCD**DQSLGGRPETVAESLSFALTA
IDPVYLPSPFDKELLKQQMCDPVIRGLLAYMKRVAQFCTPSQVARVRQITIAMFVTMAAEGPWRLYGTQPTVAEYLA
SRQVNSFWPCLVLIDLIGGYEVPANTYSRPDIHHVTALASLATTLV**NDLYSAYKE**HLNETGDFKLPYLLAARHNCSL
QEAIDLAADIHDAVMEEYERLHATLMKGTRSPVLRRYLTGLSTWIGGNLE**W**HKHSA**RY**HI

> spiroalbatene synthase (WP_030426588.1), *Allokutzneria albata*
MPKLGLSALVPGFTEPSTPPVNPLAERAEDEVVAWLWRIGFLTSQAQEQHLRSFRFGLYHGIATPELDLPALVLGMK
WFCWGSLA**DDQYD**NYDWGDRDARMRSVIRSARTILGGGAVPRTDPVIRGLAEFWPSLVAGMSPAARRRVTRNFLDYL
DAVRFQNRFHAKGDIPDAATFLGLRRHTIAMIFQADVLEALSSLDIPAVLRGHRMFRELVSCFADITAWH**NDVYGLE**
KDIADGQLCNTVLVVSAGEECSTEVAVSRVVERAKERQRLFLGIEAELPWLAEELGLGPEAVASALVLTRQLRAYAY
ANLV**W**IGQTR**RY**DLDLPRIRGTFDDVLCDG

Supplementary Figure 5. Protein sequences of diterpene TPS from different bacteria with accession numbers in parenthesis. Highlighted are the Mg^{2+} binding motifs in red and yellow as well as the WXXXXXRY motif in light blue. Underlined sequences refer to crystal structures of the respective diterpene TPS in the closed conformation, with a structured C-terminus.

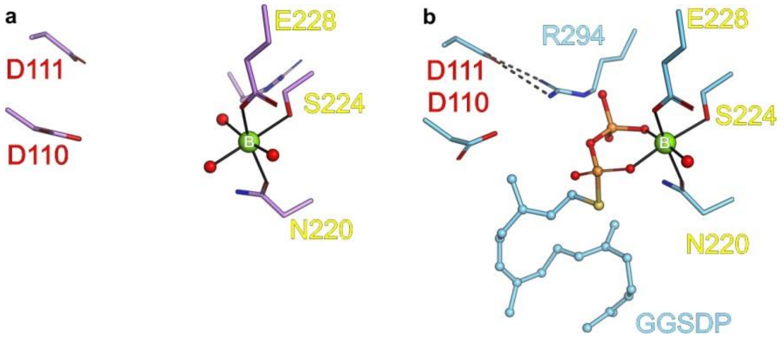

Supplementary Figure 6. CotB2 in different catalytic states. Residues of the DDXD motif are labelled with red letters, residues of the NSE motif with yellow letters, respectively. Mg^{2+}-ions are depicted as green spheres and water molecules as red spheres. Hydrogen bonds are indicated by dashed lines. Solid lines represent the coordination sphere of the Mg^{2+}-ions. **a,** Catalytic centre of $CotB2^{F107A}$•Mg^{2+}_{B}. **b,** Catalytic centre of $CotB2^{wt}$•Mg^{2+}_{B}•GGSDP.[3]

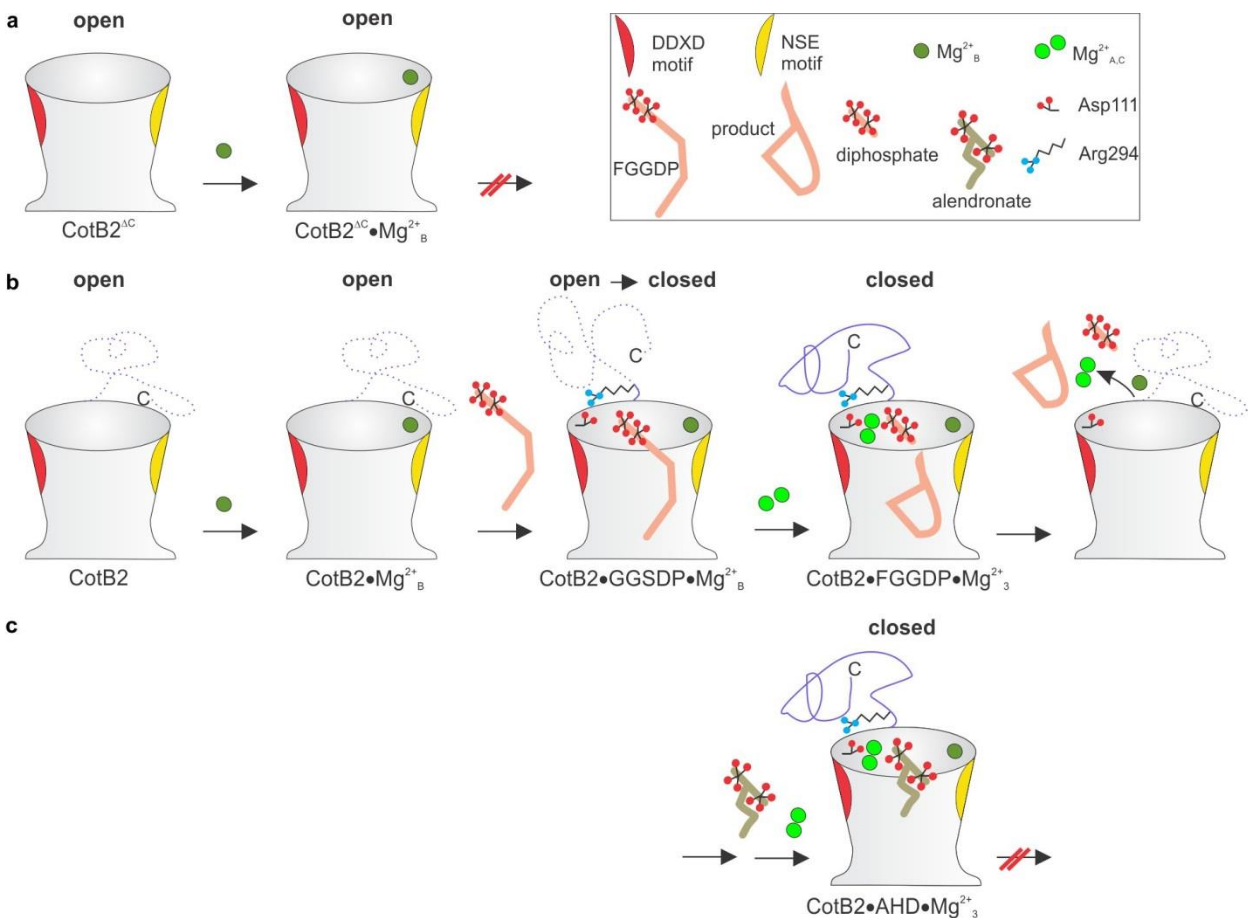

Supplementary Figure 7. Schematic drawing of the different structural snapshots of CotB2. CotB2 is schematically drawn as a gray cup. The Mg^{2+} binding motifs are indicated in red (^{110}DDXD^{113} motif) and yellow (^{218}NSE^{228} motif). The unfolded C-terminus of the open state of CotB2 is indicated by a dashed line and in the folded, closed state as a solid purple line. **a,** CotB2$^{\Delta C}$ is still capable of binding to Mg^{2+}_B (dark green circle) by the NSE motif (relevant structure CotB2$^{\Delta C}$•Mg^{2+}_B; PDB-ID 6GGK). But the missing C-terminus does not allow neither to bind nor to cyclize the educt. **b,** In the open structure of CotB2 the C-terminus is not folded (relevant structure CotB2^{wt}; PDB-ID 4OMG ref[10]). As the first binding event, Mg^{2+}_B is bound by the NSE motif (relevant structure CotB2^{F107A}•Mg^{2+}_B; PDB-ID 6GGL). Subsequently the substrate GGDP and the C-terminus starts to fold over the active site. A salt-bridge between R294 and D111 of the DDXD motif is at this stage already established (relevant structure CotB2^{wt}•Mg^{2+}_B•GGSDP; PDB-ID 5GUE ref[3]), but most of the C-terminus remains unfolded. Binding of $Mg^{2+}_{A,C}$ (light green circles) to the DDXD motif finally leads to a fully structured C-terminus that now completely closes the active site. Now, CotB2 is ready to initiate the cyclisation reaction and to cleave the diphosphate moiety (relevant structure CotB2^{wt}•Mg^{2+}_3•F-Dola; PDB-ID 6GGI). Finally the cyclised product is released. **c,** Alendronate (AHD) is recognised by CotB2 like the substrate-analogue FGGDP, but the diphosphate

moiety cannot be cleaved (relevant structure CotB2^{wt}•Mg^{2+}_3•AHD; PDB-ID 6GGJ). The diphosphate moiety of FGGDP and AHD occupies an identical position in both structures. The C-terminus is folded as in the closed structure of CotB2^{wt}•Mg^{2+}_3•F-Dola. Since no cyclisation occurs, the AHD remains captured in the active site.

a

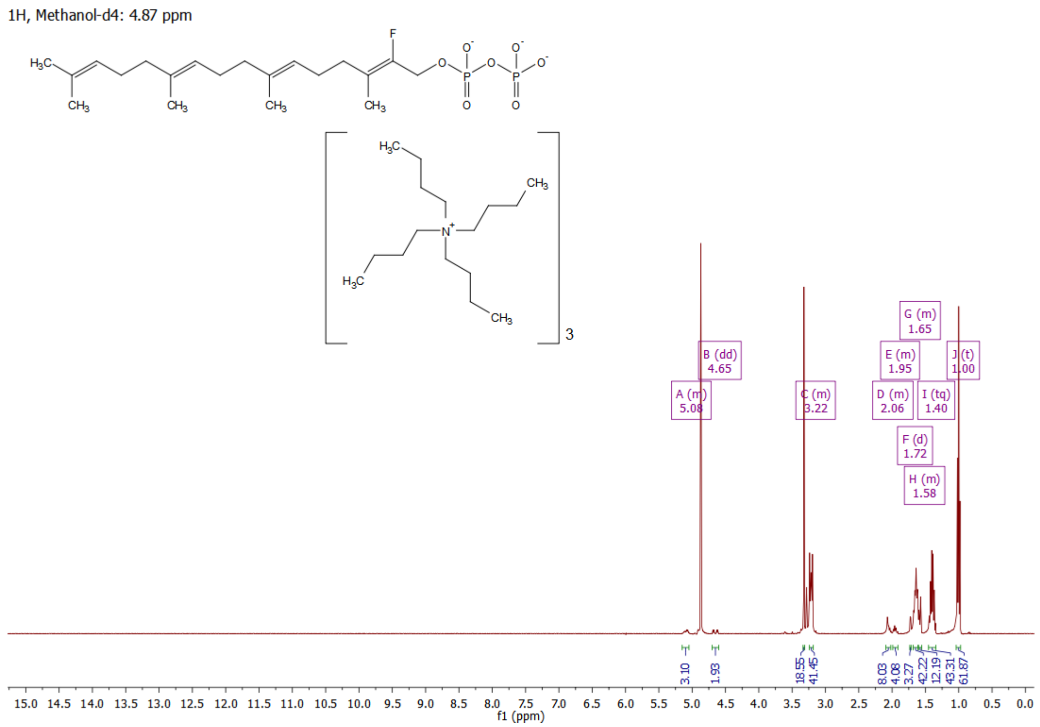

b

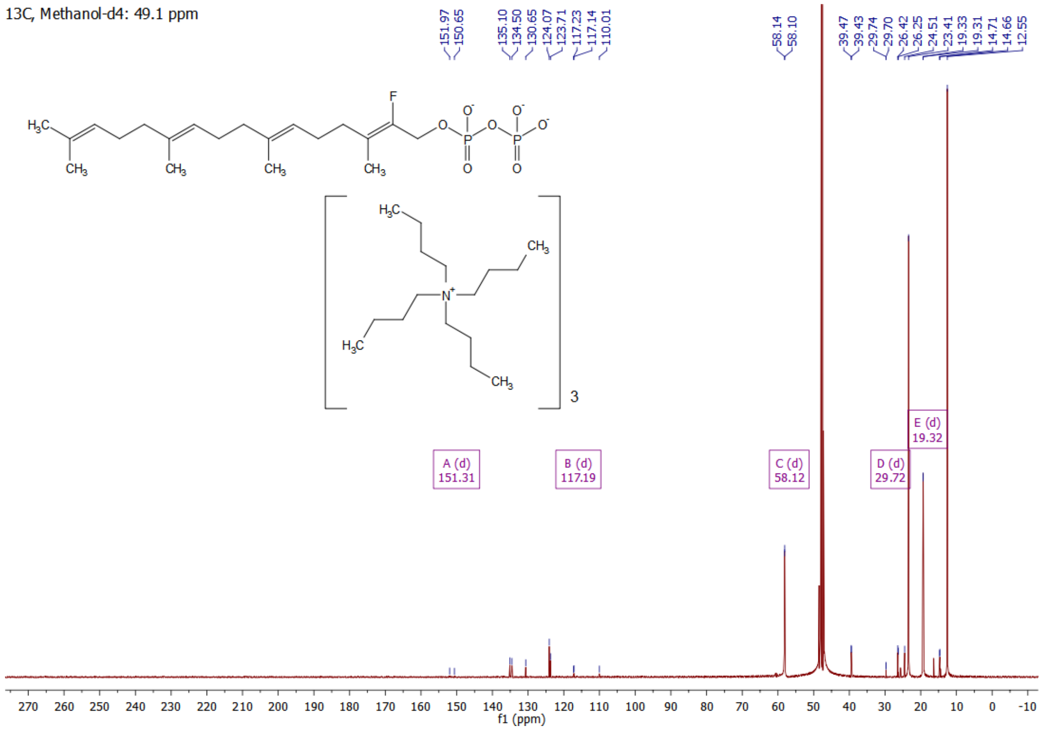

Supplementary Figure 8. NMR analysis of the tetrabutylammonium salt of 2-fluoro-geranylgeranyl diphosphate. a, ^{1}H-NMR **b,** ^{13}C-NMR.

a

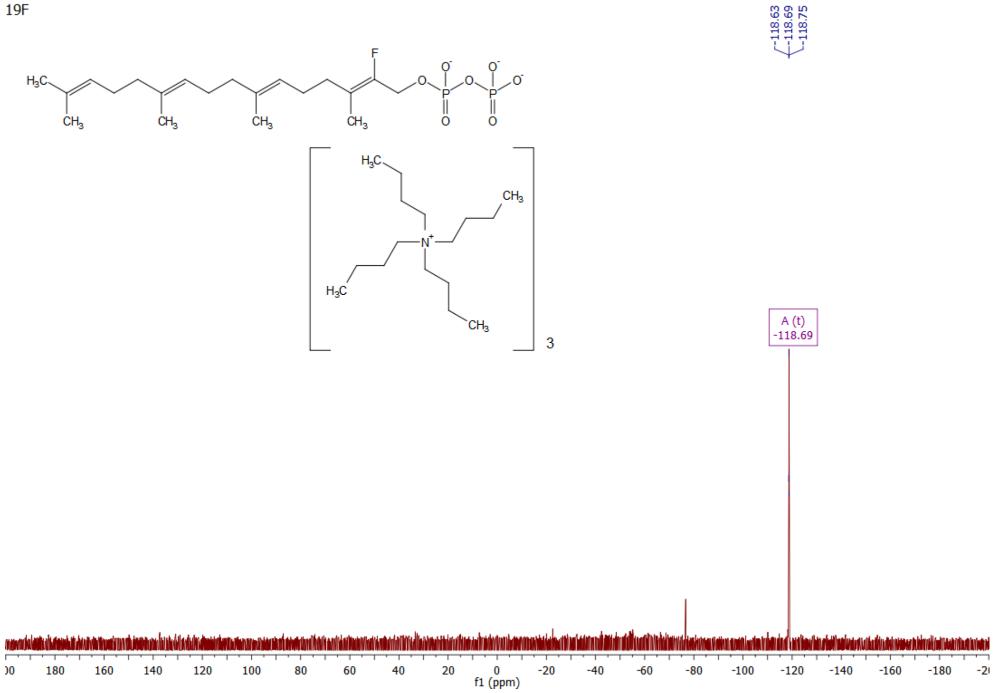

b

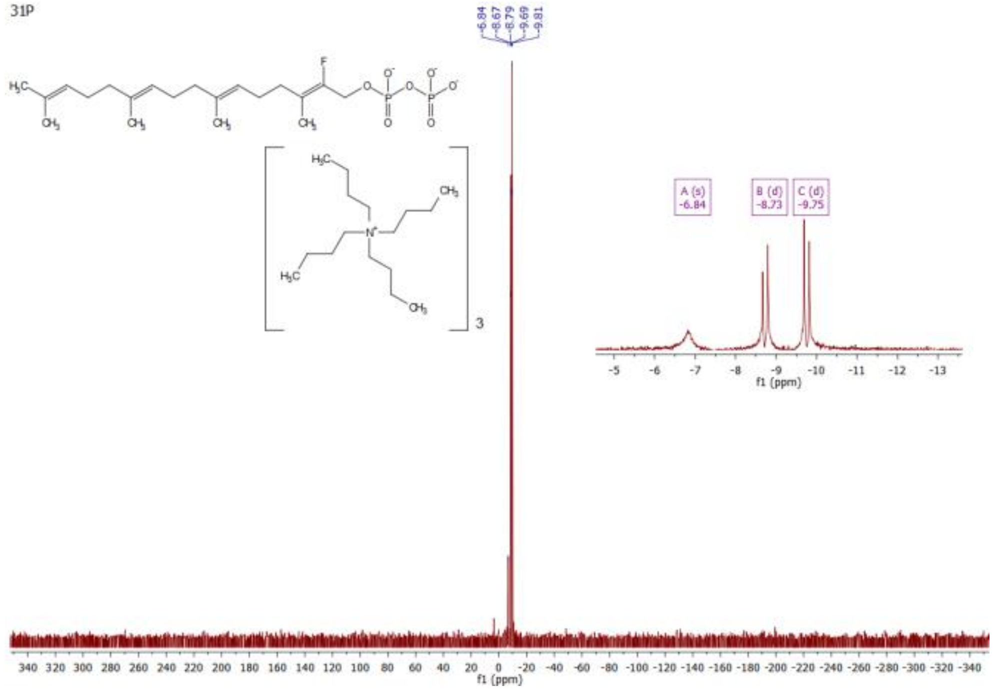

Supplementary Figure 9. NMR analysis of the tetrabutylammonium salt of 2-fluoro-geranylgeranyl diphosphate. a, ^{19}F-NMR **b,** ^{31}P-NMR.

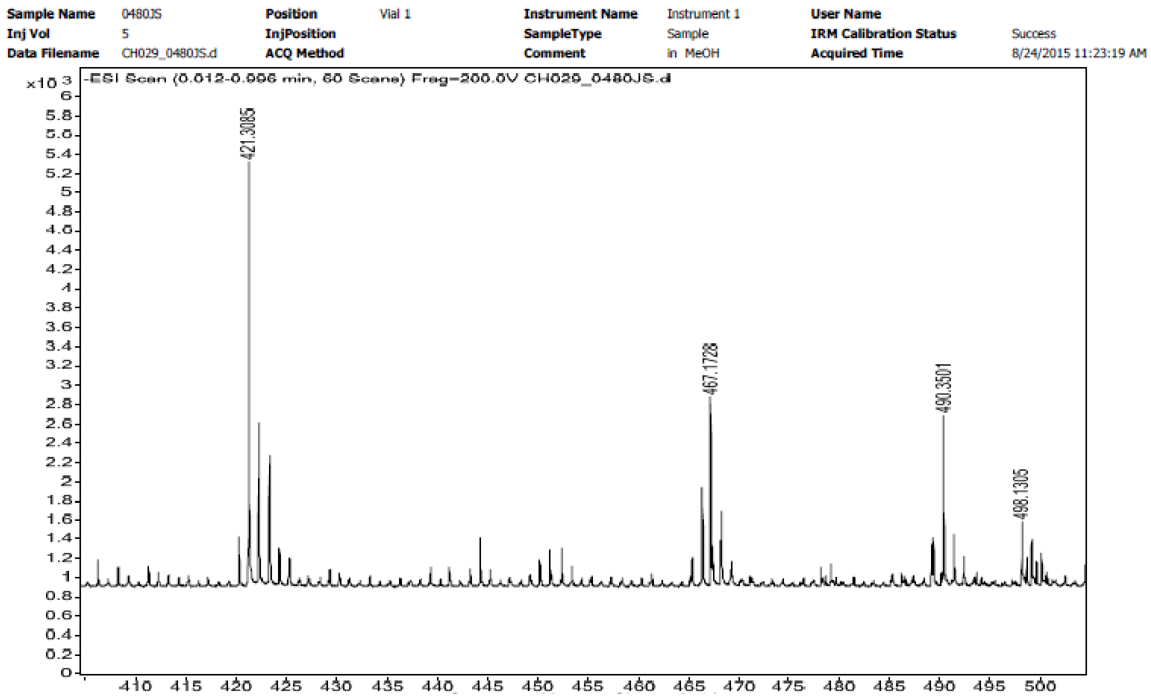

Supplementary Figure 10. HRMS analysis of tetrabutylammonium salt of 2-fluoro-geranylgeranyl diphosphate. (ESI-TOF, *m/z*): ($C_{20}H_{34}FO_7P_2^-$) $[M-H]^-$ = 467.1728 (calculated 467.1768).

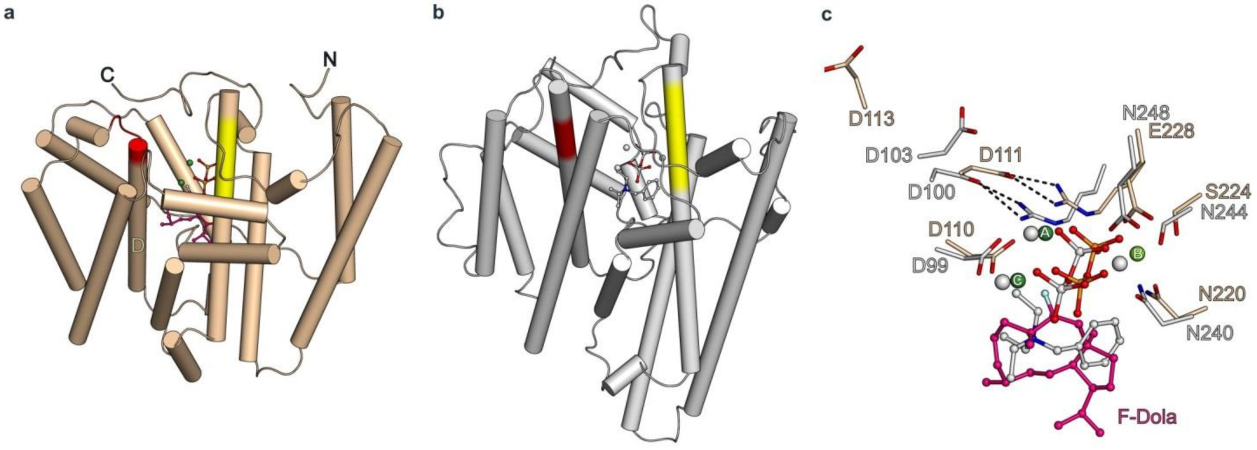

Supplementary Figure 11. The non-canonical DDXD motif in comparison to other TPSs with the canonical DDXXD motif. a. The overall structure of $CotB2^{wt}{\bullet}Mg^{2+}_3{\bullet}$F-Dola with α-helices drawn as cylinders. The ^{110}DDMD^{113} motif residing on helix D is highlighted in red and the NSE motif in yellow **b.** The overall structure of epi-isozizaene synthase (PDB ID 3KB9 ref[2]) with α-helices drawn as cylinders. The ^{99}DDRHD^{103} motif is highlighted in red and the DTE motif in yellow. **c.** Superposition of the catalytic motifs in $CotB2^{wt}{\bullet}Mg^{2+}_3{\bullet}$F-Dola and epi-isozizaene synthase. Zoom into the active site. Same view as in a and b. The Mg^{2+} in the $CotB2^{wt}{\bullet}Mg^{2+}_3{\bullet}$F-Dola structure are drawn as green spheres and of epi-isozizaene synthase as gray spheres. The bound F-Dola is shown in magenta and the bound N-benzyl-N,N-diethylethanaminium in gray stick representation, respectively. For clarity water molecules have been omitted.

Supplementary References

1 Koksal, M., Jin, Y., Coates, R. M., Croteau, R. & Christianson, D. W. Taxadiene synthase structure and evolution of modular architecture in terpene biosynthesis. *Nature* **469**, 116-120, (2011).

2 Aaron, J. A., Lin, X., Cane, D. E. & Christianson, D. W. Structure of epi-isozizaene synthase from Streptomyces coelicolor A3(2), a platform for new terpenoid cyclization templates. *Biochemistry* **49**, 1787-1797, (2010).

3 Tomita, T. *et al.* Structural Insights into the CotB2-Catalyzed Cyclization of Geranylgeranyl Diphosphate to the Diterpene Cyclooctat-9-en-7-ol. *ACS Chem. Biol.* **12**, 1621-1628, (2017).

4 Roe, S. J., Oldfield, M. F., Geach, N. & Baxter, A. A convergent stereocontrolled synthesis of [3-(14) C]solanesol. *J. Labelled Comp. Radiopharm.* **56**, 485-491, (2013).

5 Long, J. *et al.* Total syntheses of parthenolide and its analogues with macrocyclic stereocontrol. *J. Med. Chem.* **57**, 7098-7112, (2014).

6 Cane, D. E., Yang, G., Xue, Q. & Shim, J. H. Trichodiene synthase. Substrate specificity and inhibition. *Biochemistry* **34**, 2471-2479, (1995).

7 Görner, C., Hauslein, I., Schrepfer, P., Eisenreich, W. & Brück, T. Targeted Engineering of Cyclooctat-9-en-7-ol Synthase: A Stereospecific Access to Two New Non-natural Fusicoccane-Type Diterpenes. *Chemcatchem* **5**, 3289-3298, (2013).

8 Ajikumar, P. K. *et al.* Isoprenoid pathway optimization for Taxol precursor overproduction in Escherichia coli. *Science* **330**, 70-74, (2010).

9 Kim, S. Y. *et al.* Cloning and heterologous expression of the cyclooctatin biosynthetic gene cluster afford a diterpene cyclase and two p450 hydroxylases. *Chemistry & biology* **16**, 736-743, (2009).

10 Janke, R., Görner, C., Hirte, M., Brück, T. & Loll, B. The first structure of a bacterial diterpene cyclase: CotB2. *Acta Crystallogr. D Biol. Crystallogr.* **70**, 1528-1537, (2014).

11 Holm, L. & Rosenstrom, P. Dali server: conservation mapping in 3D. *Nucleic Acids Res* **38**, W545-549, (2010).

12 Tomita, T. *et al.* Structural Insights into the CotB2-Catalyzed Cyclization of Geranylgeranyl Diphosphate to the Diterpene Cyclooctat-9-en-7-ol. *ACS chemical biology* **12**, 1621-1628, (2017).

13 Shishova, E. Y., Di Costanzo, L., Cane, D. E. & Christianson, D. W. X-ray crystal structure of aristolochene synthase from Aspergillus terreus and evolution of templates for the cyclization of farnesyl diphosphate. *Biochemistry* **46**, 1941-1951, (2007).

14 Baer, P. *et al.* Induced-fit mechanism in class I terpene cyclases. *Angew Chem Int Ed Engl* **53**, 7652-7656, (2014).

15 Serrano-Posada, H. *et al.* Crystallization and X-ray diffraction analysis of a putative bacterial class I labdane-related diterpene synthase. *Acta Crystallogr F Struct Biol Commun* **71**, 1194-1199, (2015).

16 Harris, G. G. *et al.* Structural Studies of Geosmin Synthase, a Bifunctional Sesquiterpene Synthase with alpha alpha Domain Architecture That Catalyzes a Unique Cyclization-Fragmentation Reaction Sequence. *Biochemistry* **54**, 7142-7155, (2015).

17 Chen, M., Chou, W. K., Toyomasu, T., Cane, D. E. & Christianson, D. W. Structure and Function of Fusicoccadiene Synthase, a Hexameric Bifunctional Diterpene Synthase. *ACS chemical biology* **11**, 889-899, (2016).

18 Rynkiewicz, M. J., Cane, D. E. & Christianson, D. W. Structure of trichodiene synthase from Fusarium sporotrichioides provides mechanistic inferences on the terpene cyclization cascade. *P Natl Acad Sci USA* **98**, 13543-13548, (2001).

19 Koksal, M., Chou, W. K., Cane, D. E. & Christianson, D. W. Unexpected reactivity of 2-fluorolinalyl diphosphate in the active site of crystalline 2-methylisoborneol synthase. *Biochemistry* **52**, 5247-5255, (2013).

20 Liu, W. *et al.* Structure, function and inhibition of ent-kaurene synthase from Bradyrhizobium japonicum. *Sci Rep* **4**, 6214, (2014).
21 Liebschner, D. *et al.* Polder maps: improving OMIT maps by excluding bulk solvent. *Acta Crystallogr. D Biol. Crystallogr.* **73**, 148-157, (2017).
